# An analytical model of population level chronic conditions and COVID-19 related hospitalization in the United States

**DOI:** 10.1186/s12889-022-12531-3

**Published:** 2022-02-01

**Authors:** Biplab K. Datta, Benjamin E. Ansa, Varghese George

**Affiliations:** 1 Department of Population Health Sciences, Medical College of Georgia at Augusta University, Augusta, GA USA; 2 Institute of Public and Preventive Health, Augusta University, Augusta, GA USA

**Keywords:** Chronic conditions, COVID-19, Hospitalization, ICU admission, Hypertension, Diabetes, CVD

## Abstract

**Background:**

The surge in the COVID-19 related hospitalization has been straining the US health system. COVID-19 patients with underlying chronic conditions have a disproportionately higher risk of hospitalization and intensive care unit (ICU) admission. We developed a retrospective analytical model of COVID-19 related hospitalizations and ICU admissions linked to each of the three major chronic conditions – hypertension, diabetes, and cardiovascular diseases (CVD).

**Methods:**

Based on the differential probability of hospitalization of the COVID-19 patients with and without a chronic condition, we estimate a baseline cumulative hospitalization rate and ICU admission rate using the population level chronic condition prevalence from the 2019 Behavioral Risk Factor Surveillance System survey. Next, we estimate the hospitalization and ICU admission rates under an alternative scenario of a lower prevalence of the same chronic condition, aligned with the World Health Organization target of 25% relative reduction of prevalence by 2025. We then compare the outcomes of the baseline and the alternative scenarios.

**Results:**

We estimate that the lower prevalence of hypertension would have lowered the cumulative hospitalization and ICU admission rates by more than 2.5%. The lower prevalence of diabetes and CVD would lower the cumulative hospitalization rate by 0.6% and 1.4% respectively. The decrease in the rates would have been relatively higher among Black and elderly (age 55+).

**Conclusions:**

Our model, thus, provides evidence on the importance of prevention, control, and management of chronic conditions to lessen the overwhelming financial and public health burden on the health system during a pandemic like the COVID-19.

## Background

Apart from cancer, heart disease and diabetes are the leading causes of deaths and disabilities in the United States (US) population [1]. Heart disease, stroke and other cardiovascular diseases (CVD), for which hypertension is a major risk factor, account for nearly one-third of all deaths in the US [2]. One in every three US adults (age 18+) have hypertension and about 13% have diabetes [2, 3]. The risk of COVID-19 mortality is significantly greater among those with CVD, hypertension, and diabetes [4] and the population level prevalence rates of these chronic conditions are linked to COVID-19 related hospitalization, which is also higher among patients with these underlying conditions [5].

Studies on the COVID-19 related hospitalization mostly examined the odds of hospitalization and mortality among hospitalized patients by demographic characteristics such as age, sex, race, ethnicity, etc., and underlying health conditions [6–11]. While these studies provide important insights on the association between COVID-19 related hospitalization and health conditions by assessing the demographic, social and health determinants of hospitalization, there is a dearth of analysis on how progress in the population level management of chronic diseases would have lessened the burden of COVID-19 related hospitalization.

The health systems in many communities are overwhelmed by the surge in COVID-19 cases resulting in higher demands for hospital beds, medical staff, and supplies needed to provide adequate care for both COVID-19 and non- COVID-19 patients. The intensive care units (ICU) are also overwhelmed from the increasing demands for resources necessary for patient care. Patients with other conditions who normally should have been admitted to the ICU may be replaced for seriously ill COVID-19 patients. Hospitals reaching near capacity and being overflowed with COVID-19 patients thereby pose a big concern to the health system. Given the strong association between the underlying chronic health conditions and COVID-19 related hospitalizations, a question naturally arises about by what magnitude the hospitalization and ICU admission rates related to COVID-19 would have been lowered if the prevalence of some chronic conditions were lower than the status quo. This study aimed to answer this question by developing an analytical model of COVID-19 related hospitalizations linked to the population level prevalence of some chronic conditions.

Investigating this issue has important public health implications. The estimated public health burden of chronic conditions often motivates policies to effectively manage chronic diseases and thereby improving population health [12]. Quantifying the extent of hospitalization and ICU admission that could have been averted from a lower (than status quo) chronic condition prevalence, therefore, may convey an important message for strategizing effective interventions. In doing so, we separately linked three major chronic conditions (hypertension, diabetes, and CVD) in the US with COVID-19 related hospitalizations and approximately replicate the cumulative hospitalization rate for the period March 07, 2020 to January 02, 2021, reported by the Centers for Disease Control and Prevention (CDC). We then examined what could have been the hospitalizations and subsequent ICU admissions under a lower prevalence of the respective chronic conditions in adult (age 18+) population. Understanding this concept is important as it underscores the prevention and control of chronic diseases for reducing the burden of healthcare systems during a pandemic. The findings of this study contribute to existing efforts for effective management of chronic diseases at the population level and to streamline health system preparedness for managing similar pandemics.

## Methods

### Model framework

We develop a retrospective analytical model of COVID-19 related hospitalization and subsequent ICU admission for the US adult population, as a function of the prevalence of a given chronic condition. Using our model, we estimate the hospitalization and ICU admission rate per 100,000 population, specific to three major chronic conditions in US population – hypertension, diabetes, and CVD. The model links the prevalence of a chronic condition by state, race, and age-group, with the number of hospitalization and ICU admissions across these groups. The results are driven by the variations in model parameters across the state, race, and age-group.

The model follows a simple framework where a portion of the population is tested for COVID-19, a portion of the COVID-19 positive patients gets admitted in the hospital, and a portion of the hospitalized COVID-19 patients receives ICU care. Several assumptions, supported by data and the findings from existing literature, are used within this framework to replicate the COVID-19 related cumulative hospitalization rates among adults in the USA from the week ending March 07, 2020 to the week ending January 02, 2021. We begin with the assumption that the probability of being tested for COVID-19 varies across states. For example, someone residing in New York has a larger probability of receiving a COVID-19 test compared to someone residing in Kentucky [13]. Second, the probability of COVID-positivity among those who receive a test varies across race and age-group. This is because of the disproportionate risk of exposure associated with the social and economic factors such as occupation and housing [14]. Third, among the patients tested COVID-positive, the rate of hospitalization varies by age-group and whether the patient has an underlying chronic condition or not [5]. Fourth, the presence or absence of the chronic condition is associated with individual’s age-group, race, and state of residence [15]. Stated differently, the prevalence of certain chronic condition varies across state, race, and age-group. Finally, the probability of ICU admission also varies across age-group [16] and race.

**Fig. 1:**
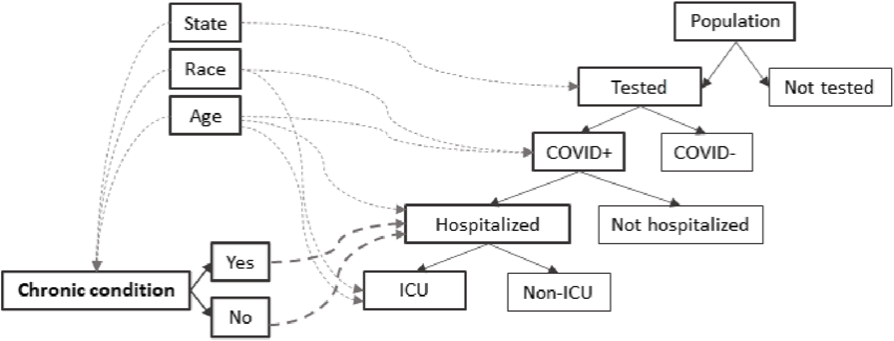
Model framework. The solid lines (with arrowheads) represent flow from one stage to subsequent stage within the framework (e.g., from being COVID positive to hospitalized). The dashed lines (with arrowheads) represent probabilistic variation due to demographic (i.e., state of residence, race, and age) and health (i.e., hypertension, diabetes, or CVD) factors (e.g., probability of being COVID positive varies by race and rage)

Based on these assumptions, the flow of the model is as follows: A sample from the state’s adult population is tested for COVID-19, a subset of those tested positive are hospitalized, and a subset among the hospitalized patients are admitted in the ICU (Fig. 1). The primary mechanism of the model is that whether a COVID-19 patient would require hospitalization depends on whether that patient has the underlying chronic condition or not. This essentially entails the assumption that a COVID-19 patient is provided with hospital care as deemed needed (and prioritized based on health condition) by the physicians. The model is separately implemented for the three chronic conditions. The adult population in the model is divided in six age groups – 18-24, 25-34, 35-44, 45-54, 55-64 and 65+; and four racial categories – White, Black, Hispanic, and Other (American Indian, Asian, Native Hawaiian/ Pacific Islander, multiracial and other), across the 50 US states and the District of Columbia.

### Model calculation

Let *H*_*s*,*r*,*a*_ be the number of COVID-19 related hospitalization and *ICU*_*s*,*r*,*a*_ be the number of ICU admissions among individuals of race *r* and age-group *a* in state *s*. The hospitalization and ICU admission rates are defined as follows:

1 $$ \begin{aligned} & \frac{H_{s,r,a}}{pop_{s,r,a}} = t_{s}*v_{r,a}*[c_{s,r,a}*h_{c,a}+(1-c_{s,r,a})*h_{noc,a}]*100,000 \end{aligned}  $$

2 $$ \begin{aligned} & \frac{ICU_{s,r,a}}{pop_{s,r,a}} = i_{r,a}*t_{s} *v_{r,a} * [c_{s,r,a}*h_{c,a}+(1-c_{s,r,a})*h_{noc,a}]*100,000 \end{aligned}  $$

where, *pop*_*s*,*r*,*a*_ is the population size of race *r* and age-group *a* in state *s*, *t*_*s*_ is the probability of being tested in state *s*, *v*_*r*,*a*_ is the probability of COVID-positivity for race *r* and age-group *a*, *c*_*s*,*r*,*a*_ is the prevalence of chronic condition *c* among race *r* and age-group *a* in state *s*,*h*_*c*,*a*_ is the probability of hospitalization for individuals of age-group *a* with chronic condition *c*, *h*_*noc*,*a*_ is the probability of hospitalization for individuals of age-group *a* without the chronic condition *c*, and *i*_*r*,*a*_ is the probability of ICU admission for race *r* and age-group *a*. Total COVID-19 related hospitalization and ICU admissions at certain level (age-group and race or any combination) are obtained by aggregating *H*_*s*,*r*,*a*_ and *ICU*_*s*,*r*,*a*_ over appropriate levels, i.e., age-groups, race, and/or state.

### Data

Estimating the model requires population and chronic condition prevalence data by state, race, and age-group. We used bridged-race postcensal population estimates of 2019 from the National Vital Statistics Systems [17]. The status quo chronic condition prevalence data were obtained from the 2019 Behavioral Risk Factor Surveillance System (BRFSS) data [18].

Data on the number of COVID-19 tests performed in age 18+ population across states were obtained from the Center for Disease Control and Prevention’s (CDC) *COVIDView* and *CDC COVID Data Tracker* websites [19, 20]. Data on the number of COVID-19 positive cases as of December 26, 2020 and age distribution of the cases were obtained from the *CDC COVID Data Tracker* website [20]. The probabilities of COVID-positivity by race and age-group were calculated using estimates from Rentsch et al. (2020) [21] and Vahidy et al. (2020) [22], two studies providing complementary information on the racial and ethnic characteristics of COVID-19 test recipients in the USA by age groups.

**Fig. 2:**
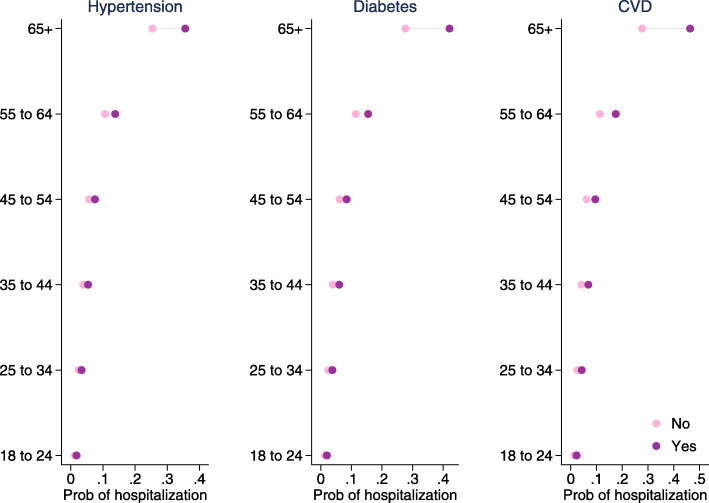
Hospitalization probability by age-group and underlying chronic condition. Source: Authors’ calculation from Yehia et al. (2020) and CDC data (The COVID-19-Associated Hospitalization Surveillance Network hospitalization data). The following formula is used to obtain probability of hospitalization by age for those not having chronic condition *c*: $p_{a} (hospitalization|noc)=1+\left [1-\frac {p(hospitalization|c))}{p(hospitalization|noc)}\right ]*p_{a} (hospitalization|COVID) *scalefactor_{c,a}$. A chronic-condition-age specific scale factor is included in the equation for adjustments in the probabilities that culminate in replication of the reported cumulative hospitalization rate. The following formula is used to obtain probability of hospitalization by age for those having chronic condition *c*: $p_{a} (hospitalization|c)=p_{a} (hospitalization|noc)*\frac {p(hospitalization|c))}{p(hospitalization|noc)}$

**Fig. 3:**
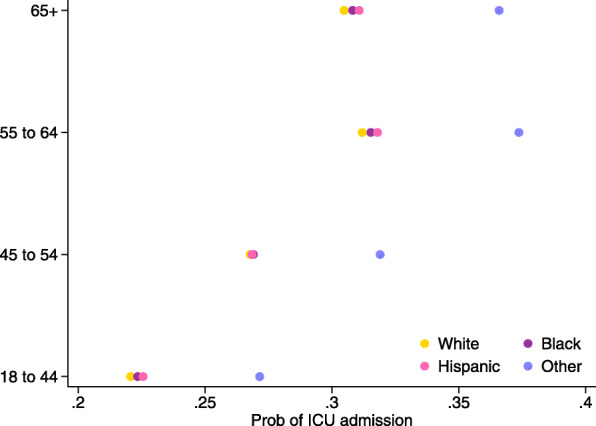
ICU admission probability by age-group and race. Source: Authors’ calculation from the CDC data (The COVID-19-Associated Hospitalization Surveillance Network hospitalization data). The following formula is used to obtained the conditional probabilities: $P(ICU|Race \& Age)=\frac {P(Race|ICU)P(Age|ICU)P(ICU)}{P(Race|ICU)P(Age|ICU)P(ICU)+P(Race|NonICU)P(Age|NonICU)P(NonICU)}$

The cumulative COVID-19 hospitalization rates by age-group as of January 02, 2021 were obtained from the CDC’s *COVID-NET* website [23]. Hospitalization per positive case was calculated by dividing the cumulative hospitalization rate per 100,000 adult population by the cumulative incidence rate per 100,000 adult population. Probabilities of hospitalization by chronic condition were calculated using the estimates from Yehia et al. (2020) [24], adjusted by the hospitalization per positive case for respective age-groups. Figure 2 shows the hospitalization probabilities that were used in the model estimation, by age-group and with and without the chronic condition. The ICU admission data were obtained from the CDC *COVID-NET* website [23]. ICU admission probabilities by race and age-group were calculated using conditional probability with multiple conditions. Figure 3 shows the ICU admission probabilities that were used in the model estimation, by age-group and race.

### Model estimation

We first estimated a baseline for each of the three chronic conditions – hypertension, diabetes, and CVD, using the status quo level of prevalence (i.e., actual prevalence in 2019) of respective condition. The baseline estimates (for hypertension, diabetes, and CVD) replicate the cumulative hospitalization rate from March 07, 2020 to January 02, 2021 as reported in the *COVID-NET* data. Next, we estimated an alternative scenario of a lower prevalence, meaning number of adults with chronic condition be lower the that in the status quo, for each of the three chronic conditions. The World Health Organization, in 2013, adopted a global action plan to reduce the “preventable and avoidable" burden of noncommunicable diseases (NCDs) by 2025. The plan outlined voluntary targets such as a 25% relative reduction in premature mortality from NCDs including CVD and diabetes, and a 25% relative reduction in the prevalence of raised blood pressure or hypertension from the 2010 (baseline) level [25]. The prevalence trajectory, aiming the relative reduction target in 2025, serves as an alternative prevalence scenario for assessing what could have been the hospitalization and ICU admission rates under the lower prevalence rates. Though no prevalence-reduction target was set for diabetes and CVD, in the same vein as for the hypertension, we considered a 25% relative reduction in prevalence for these two conditions.

**Fig. 4:**
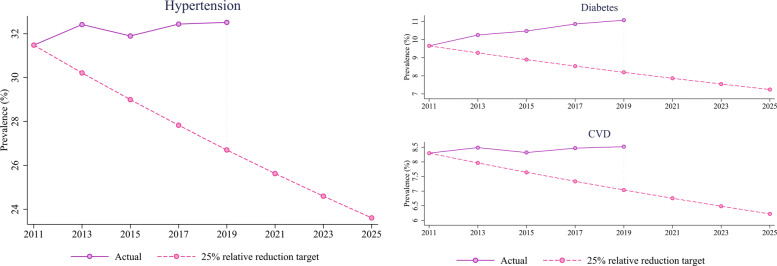
Actual and alternative prevalence trend. The 2025-target prevalence is 75% of the prevalence in 2011. Compound biannual growth rate was used to construct the 25% relative reduction target trajectory from 2011 to 2025

Figure 4 illustrates the prevalence rates of hypertension, diabetes, and CVD in the US adult (18+) population. The alternate hypertension prevalence (on the 25% relative reduction target trajectory) in 2019 would have been 26.71%, which is 17.87% lower than the actual prevalence of 32.52%. The alternate prevalence rates of diabetes and CVD would have been 8.20% and 7.04% respectively, which are 26.03% and 17.35% lower than the actual rates in 2019 (Fig. 4). After estimating the baseline, we re-estimate the model using these alternate prevalence rates for respective chronic conditions. We report the hospitalization and ICU admission rate per 100,000 adult population in the baseline and the differences in rates between the alternative scenario and the baseline. A negative difference refers to a decrease from the baseline. We report the results by race and age-group aggregated over the states. We also report the differences in hospitalization and ICU admission rates by state.

## Results

During the year 2020 (from March 7, 2020 to January 2, 2021), the cumulative hospitalization rate (in 18+ population) per 100,000 adults in the COVID-NET database was 466.7 [23]. Our baseline estimates of hospitalization rate (for hypertension, diabetes, and CVD) approximately replicates this reported rate. Table 1 presents the baseline hospitalization rate estimates by race and age-group and the change in hospitalization rate under the alternative scenario. For the hypertension scenario, had there been 17.87% less hypertensive adults (than status quo), the hospitalization rate would have been lowered by about 12 points (2.6%). The biggest changes would have occurred for the older age-groups (55 and above) and for the Black population. The findings are similar for the diabetes and CVD scenarios as well, though in a lesser magnitude. Had there been 26.03% less adults with diabetes (than status quo), the overall cumulative hospitalization rate would have been 0.6% lower. However, among the non-White population, the rates would have been more than 2% lower. In case of CVD, had the prevalence been 17.35% lower (than status quo), the overall cumulative hospitalization rate would have been 1.4% lower.

**Table 1:** Cumulative hospitalization rate estimates

	Hospitalization Rate (per 100,000)					*Δ* Hospitalization Rate (per 100,000)				
	White	Black	Hispanic	Other	All	White	Black	Hispanic	Other	All
Hypertension										
18 to 24	60	136.3	153.5	65.7	93.1	-0.3	-0.7	-0.8	-0.3	-0.5
25 to 34	115	265.4	295	124.9	175.3	-1.1	-2.7	-2.1	-0.7	-1.5
35 to 44	173.4	412.8	448.2	267.3	269.7	-2.3	-7.4	-4.9	-3.3	-3.6
45 to 54	226.7	555	603.2	399.8	346.9	-3.8	-13.1	-9.3	-5.6	-6.1
55 to 64	367.9	921.5	1012.2	771.8	534.6	-7.5	-26.7	-21.1	-15.5	-11.9
65+	814.4	2041.3	2249	1992.6	1115.2	-27.4	-83.9	-76.1	-67.1	-39
All	359.4	700.2	658.3	582.7	467.1	-9.5	-21.4	-14.2	-14.7	-12.1
%-change						-2.6	-3.1	-2.2	-2.5	-2.6
Diabetes										
18 to 24	60.3	137.6	154.8	66.3	93.8	-0.1	-0.3	-0.2	-0.1	-0.1
25 to 34	113.4	262.4	296.2	125.5	174.3	-0.2	-1	-1.1	-0.2	-0.5
35 to 44	169	397.4	449.2	263.9	265	-0.9	-3.7	-3.9	-1.8	-2
45 to 54	230	552	632.8	412.6	354.7	-2	-8.1	-9.5	-3.6	-4.2
55 to 64	367.5	904.3	1045.4	786.6	537.1	-4.6	-19.7	-22.3	-13.3	-9
65+	793	2016.2	2356.9	2007.4	1106.4	-19.1	-79.4	-96.8	-64.3	-33.8
All	353.5	690	679.9	588.8	466.1	-6.2	-17.7	-16.1	-13.2	-3
%-change						-1.7	-2.6	-2.4	-2.2	-0.6
CVD										
18 to 24	61.8	140.8	159.6	68	96.4	-0.1	-0.1	-0.3	-0.1	-0.1
25 to 34	116.3	269.5	303	129.2	178.6	-0.2	-0.8	-0.6	-0.2	-0.3
35 to 44	173	402	454.1	269.2	269.4	-0.6	-1.6	-1.5	-1	-0.9
45 to 54	227.8	542.1	611.8	408.2	348.1	-1.3	-4.6	-3.7	-2.4	-2.2
55 to 64	366	886.9	998.4	785.4	528.4	-3.7	-12.2	-9.2	-10.6	-5.8
65+	822.1	1955.3	2219.4	2014.3	1111.7	-17.6	-41.9	-40.2	-43.6	-23.2
All	361.5	678.9	658.8	591.1	466.5	-5.5	-9.7	-6.7	-9.1	-6.5
%-change						-1.5	-1.4	-1	-1.5	-1.4

The cumulative ICU admission rate estimates are presented in Table 2. Similar to the hospitalization results, the changes were larger for the older age cohorts (particularly age 65+). For hypertension and diabetes, a decline in prevalence would have resulted in a comparatively greater decline in ICU admission among the Black. For CVD, the decline, however, would have been relatively smaller (1.1%) among the Hispanic, and similar (around 1.5%) for the White, Black and other race categories.

**Table 2:** Cumulative ICU admission rate estimates

	Hospitalization Rate (per 100,000)					*Δ* Hospitalization Rate (per 100,000)				
	White	Black	Hispanic	Other	All	White	Black	Hispanic	Other	All
Hypertension										
18 to 24	13.2	30.5	34.6	17.8	21	-0.1	-0.2	-0.2	-0.1	-0.1
25 to 34	25.4	59.3	66.6	33.9	39.6	-0.2	-0.6	-0.5	-0.2	-0.3
35 to 44	38.3	92.2	101.1	72.6	61.3	-0.5	-1.7	-1.1	-0.9	-0.8
45 to 54	60.7	149.3	162	127.6	94.6	-1	-3.5	-2.5	-1.8	-1.6
55 to 64	114.8	290.6	321.9	288.4	170.8	-2.3	-8.4	-6.7	-5.8	-3.8
65+	248.2	629.1	699	729.1	348.3	-8.4	-25.9	-23.6	-24.6	-12.2
All	104.5	200.9	185.4	202.7	136.9	-2.8	-6.4	-4.2	-5.3	-3.7
%-change						-2.7	-3.2	-2.3	-2.6	-2.7
Diabetes										
18 to 24	13.3	30.7	34.9	18	21.2	0	-0.1	0	0	0
25 to 34	25	58.6	66.8	34.1	39.4	0	-0.2	-0.2	-0.1	-0.1
35 to 44	37.3	88.8	101.3	71.7	60.2	-0.2	-0.8	-0.9	-0.5	-0.4
45 to 54	61.6	148.5	169.9	131.6	96.7	-0.5	-2.2	-2.5	-1.2	-1.1
55 to 64	114.6	285.2	332.4	293.9	171.6	-1.4	-6.2	-7.1	-5	-2.9
65+	241.7	621.4	732.5	734.5	345.7	-5.8	-24.5	-30.1	-23.5	-10.6
All	102.8	198.1	191.9	204.9	136.6	-1.9	-5.4	-4.9	-4.8	-2.1
%-change						-1.8	-2.7	-2.5	-2.3	-1.5
CVD										
18 to 24	13.6	31.5	36	18.5	21.8	0	0	-0.1	0	0
25 to 34	25.7	60.2	68.4	35.1	40.4	0	-0.2	-0.1	-0.1	-0.1
35 to 44	38.2	89.8	102.4	73.1	61.2	-0.1	-0.4	-0.3	-0.3	-0.2
45 to 54	61	145.9	164.3	130.2	95	-0.4	-1.2	-1	-0.7	-0.6
55 to 64	114.2	279.7	317.5	293.5	168.8	-1.1	-3.9	-2.9	-3.9	-1.9
65+	250.6	602.6	689.8	737	347.3	-5.4	-12.9	-12.5	-16	-7.3
All	105.1	194.4	185.1	205.6	136.6	-1.7	-2.9	-2	-3.3	-2
%-change						-1.6	-1.5	-1.1	-1.6	-1.5

Table 3 presents the changes in cumulative hospitalization and ICU admission rates by state. In the case of hypertension, around 70% of the states would have more than 10 points lower cumulative hospitalization rate per 100,000 adults if hypertension prevalence were lower. The largest changes (more than 3%) would have occurred in Mississippi, Alabama, West Virginia, Louisiana, Kentucky, and Arkansas. The lowest decline would have been in Utah, Colorado, Minnesota, and California. For diabetes, the decrease in cumulative hospitalization rate would have been the largest (more than 2.5%) in Mississippi and West Virginia and the lowest in Alaska, Montana, and Vermont. Around one-fourth of the states would have experienced a more than 10 points lower cumulative hospitalization rate if the diabetes prevalence were lower. For CVD, the decrease in cumulative hospitalization rate would have been the highest for West Virginia (more than 2%) and the lowest for Colorado, Alaska, and Utah (around 1%). For lower CVD prevalence, the cumulative hospitalization rate would have been more than 5 points lower in more than 70% of the states. The changes in cumulative ICU admission rates were also similar to the changes in cumulative hospitalization rates for all three chronic conditions.

**Table 3:** Change in cumulative hospitalization and ICU admission rates by state

	Hospitalization Rate (per 100,000)						*Δ* Hospitalization Rate (per 100,000)					
	Hypertension		Diabetes		CVD		Hypertension		Diabetes		CVD	
	*Δ*	%	*Δ*	%	*Δ*	%	*Δ*	%	*Δ*	%	*Δ*	%
Alabama	-15.1	-3.2	-11.2	-2.4	-8.5	-1.8	-4.5	-3.3	-3.4	-2.5	-2.6	-1.9
Alaska	-14.2	-2.6	-6.7	-1.3	-5.4	-1	-4.5	-2.7	-2.1	-1.3	-1.7	-1.1
Arizona	-11.1	-2.5	-9.3	-2.1	-5.8	-1.3	-3.4	-2.6	-2.9	-2.2	-1.8	-1.4
Arkansas	-12.3	-3	-9.5	-2.4	-7.6	-1.9	-3.7	-3.1	-2.9	-2.4	-2.3	-1.9
California	-14	-2.4	-12.7	-2.1	-6.8	-1.1	-4.4	-2.5	-4	-2.2	-2.2	-1.2
Colorado	-7.6	-2.2	-5.1	-1.5	-3.4	-1	-2.3	-2.3	-1.6	-1.6	-1	-1.1
Connecticut	-15.5	-2.5	-11.2	-1.8	-6.8	-1.1	-4.7	-2.6	-3.4	-1.9	-2.1	-1.2
Delaware	-18.6	-2.8	-15.9	-2.3	-9.9	-1.5	-5.6	-2.8	-4.8	-2.4	-3	-1.5
DC	-19.1	-2.7	-15.8	-2.2	-9.8	-1.4	-5.8	-2.8	-4.8	-2.4	-3	-1.5
Florida	-17	-2.6	-14.7	-2.2	-9.1	-1.4	-5.2	-2.7	-4.5	-2.3	-2.8	-1.4
Georgia	-10.2	-2.7	-7.9	-2.1	-5.4	-1.5	-3.1	-2.8	-2.4	-2.2	-1.6	-1.5
Hawaii	-14.4	-2.7	-10.6	-2	-6.5	-1.2	-5	-2.8	-3.7	-2.1	-2.2	-1.3
Idaho	-6.5	-2.4	-5.3	-2	-3.6	-1.4	-1.9	-2.5	-1.6	-2.1	-1.1	-1.4
Illinois	-11.8	-2.7	-9.6	-2.2	-6	-1.4	-3.6	-2.8	-3	-2.3	-1.9	-1.5
Indiana	-9.7	-2.7	-7.9	-2.3	-5.6	-1.6	-2.9	-2.8	-2.4	-2.4	-1.7	-1.7
Iowa	-11.5	-2.5	-8.1	-1.8	-6.5	-1.4	-3.4	-2.6	-2.5	-1.9	-2	-1.5
Kansas	-10.5	-2.6	-8	-2	-5.5	-1.4	-3.1	-2.7	-2.4	-2.1	-1.7	-1.4
Kentucky	-10.9	-3	-8	-2.3	-6.7	-1.8	-3.3	-3.1	-2.4	-2.4	-2	-1.9
Louisiana	-19.1	-3	-14.7	-2.4	-9.7	-1.6	-5.7	-3.1	-4.5	-2.5	-2.9	-1.6
Maine	-8.8	-2.7	-5.6	-1.7	-5.3	-1.6	-2.6	-2.7	-1.7	-1.8	-1.6	-1.7
Maryland	-19.5	-2.7	-14.4	-2.1	-8.4	-1.2	-5.9	-2.8	-4.4	-2.2	-2.6	-1.3
Massachusetts	-13.4	-2.4	-9	-1.6	-7.4	-1.3	-4.1	-2.5	-2.8	-1.7	-2.3	-1.4
Michigan	-10.5	-2.7	-7.9	-2.1	-6	-1.6	-3.2	-2.8	-2.4	-2.1	-1.8	-1.6
Minnesota	-11.4	-2.4	-7.9	-1.6	-6.4	-1.3	-3.4	-2.4	-2.4	-1.7	-2	-1.4
Mississippi	-8.6	-3.3	-7.2	-2.8	-4.5	-1.7	-2.5	-3.3	-2.2	-2.9	-1.4	-1.8
Missouri	-5.4	-2.5	-4.2	-1.9	-3.5	-1.6	-1.6	-2.6	-1.3	-2	-1.1	-1.7
Montana	-7.4	-2.4	-4.2	-1.4	-4.6	-1.5	-2.3	-2.5	-1.3	-1.5	-1.4	-1.6
Nebraska	-10.9	-2.5	-8.1	-1.9	-6.1	-1.4	-3.3	-2.6	-2.5	-2	-1.8	-1.5
Nevada	-13.7	-2.5	-11.7	-2.1	-7.6	-1.4	-4.2	-2.6	-3.7	-2.2	-2.4	-1.4
New Hampshire	-7.1	-2.5	-4.4	-1.6	-3.6	-1.3	-2.1	-2.6	-1.3	-1.6	-1.1	-1.3
New Mexico	-15.1	-2.6	-13.6	-2.3	-7.5	-1.3	-4.6	-2.6	-4.2	-2.4	-2.3	-1.3
New York	-17.4	-2.5	-14.7	-2.1	-9.4	-1.4	-5.3	-2.6	-4.6	-2.2	-2.9	-1.4
North Carolina	-12.1	-2.8	-9.1	-2.1	-6.7	-1.5	-3.6	-2.9	-2.8	-2.2	-2	-1.6
North Dakota	-12.3	-2.5	-8.2	-1.7	-6.9	-1.4	-3.7	-2.6	-2.5	-1.8	-2.1	-1.5
Ohio	-7.7	-2.7	-6.1	-2.1	-4.6	-1.6	-2.3	-2.8	-1.9	-2.2	-1.4	-1.6
Oklahoma	-5.4	-2.9	-4.1	-2.3	-3.2	-1.7	-1.7	-3	-1.3	-2.4	-1	-1.8
Oregon	-7.8	-2.4	-4.9	-1.5	-4.4	-1.4	-2.4	-2.5	-1.5	-1.6	-1.4	-1.4
Pennsylvania	-10.9	-2.7	-7.9	-2	-6.5	-1.6	-3.3	-2.8	-2.4	-2.1	-2	-1.7
Rhode Island	-15.3	-2.6	-11.4	-2	-7.8	-1.3	-4.6	-2.7	-3.5	-2.1	-2.4	-1.4
South Carolina	-11.3	-3	-8.9	-2.4	-6.3	-1.7	-3.4	-3.1	-2.7	-2.5	-1.9	-1.7
South Dakota	-6.1	-2.5	-4.7	-2	-3.5	-1.4	-1.9	-2.6	-1.5	-2.1	-1.1	-1.5
Tennessee	-14.1	-2.9	-11.2	-2.4	-7.9	-1.6	-4.2	-3	-3.4	-2.5	-2.4	-1.7
Texas	-10.3	-2.6	-9.3	-2.3	-5.6	-1.4	-3.1	-2.7	-2.8	-2.5	-1.7	-1.5
Utah	-8.2	-2.2	-5.9	-1.6	-4	-1.1	-2.5	-2.3	-1.8	-1.7	-1.2	-1.2
Vermont	-12.4	-2.4	-7.4	-1.5	-7.2	-1.4	-3.7	-2.5	-2.2	-1.5	-2.2	-1.5
Virginia	-10.1	-2.7	-7.9	-2.1	-5	-1.4	-3.1	-2.8	-2.4	-2.2	-1.6	-1.4
Washington	-4.7	-2.4	-3.3	-1.8	-2.7	-1.4	-1.4	-2.5	-1	-1.9	-0.8	-1.5
West Virginia	-16.9	-3.1	-13.8	-2.6	-11.5	-2.1	-5.1	-3.2	-4.2	-2.7	-3.5	-2.2
Wisconsin	-11.1	-2.5	-6.7	-1.6	-5.7	-1.3	-3.3	-2.6	-2.1	-1.7	-1.8	-1.4
Wyoming	-10.4	-2.5	-6.6	-1.6	-5.8	-1.4	-3.1	-2.6	-2	-1.7	-1.8	-1.5

## Discussion

In this study, we developed an analytical model to link the population level chronic conditions with COVID-19 related hospitalization and ICU admission in the USA. Based on the differential probability of being admitted to hospital from COVID-19 related complications with and without an underlying chronic condition, the model estimates cumulative hospitalization rate and subsequent ICU admission rate per 100,000 adult population. The baseline, which replicates the cumulative hospitalization rate in the USA during the year 2020, is estimated using the status quo prevalence of the chronic condition in the US adult population. The counterfactual scenarios are estimated for a lower level (than status quo) of prevalence and compared with the baseline to illustrate what could have been the cumulative hospitalization rate had there been a lower prevalence of the chronic condition.

The model provides estimates by age group and race for three major chronic conditions in the US population – hypertension, diabetes, and CVD. The findings quantify the lower number of COVID-19 related hospitalization and subsequent ICU admission in the USA if the prevalence of the chronic conditions would have been lower. We should point out that the cumulative effect of reducing prevalence rates of multiple chronic conditions on hospitalization and ICU admission will not necessarily be additive due to the correlations and interactions among the conditions. The decrease in hospitalization and ICU admission would have been relatively higher among the non-whites, who were disproportionately affected by the pandemic. The model, thus, generates evidence on the importance of prevention, control, and management of chronic conditions to lessen the overwhelming economic and public health burden on health systems.

The risks of chronic conditions like heart diseases, hypertension and diabetes are associated with lifestyle behaviors including but not limited to smoking, unhealthy diet, lack of physical activity, and excessive alcohol consumption [1]. Modifying these risky behaviors could reduce the prevalence of chronic conditions in the population. By quantifying the magnitude of the counterfactual hospitalization and ICU admission rates, our estimates analytically document that a lower (than status quo) population level prevalence of the major chronic conditions would have reduced the stress on the health system during the COVID-19 pandemic. Our analyses thus underpin the need for an effective population level chronic disease management for avoiding undue burden on the health system.

Of note, our knowledge on the relationship between COVID-19 severity and underlying health conditions, however, are constantly evolving. Several studies report that elevated glycated hemoglobin (HbA1c) was associated with higher risk of severe COVID-19 outcomes and hospitalization [26, 27]. Other studies find that state of glycemic control after admission has critical association with outcomes in COVID-19 patients [28, 29]. Similarly, differing evidence was presented in different studies regarding the relationship between blood pressure control and outcomes among hospitalized COVID-19 patients [30–32]. Nevertheless, underlying chronic conditions are intrinsic to COVID-19 related hospitalization, and continued research will gradually shape our understanding of this critical issue.

Like other analytical models, our model has several limitations. First, based on the available information we can only model one chronic condition at a time. We do not have information on the relationship between hospitalization and multiple comorbidities. For example, we know the number of hospitalized cases having hypertension or diabetes, but do not know the number of hospitalized cases having hypertension only, or diabetes only, or both hypertension and diabetes. Therefore, we cannot distinguish between someone with hypertension only and someone with hypertension and other comorbidities. Second, the chronic condition prevalence was self-reported and not clinically diagnosed in the BRFSS data. Third, we extracted the parameter values, covering segment of population, from various studies and databases. We developed a proxy for the population level parameters with these subgroup estimates. For example, *COVID-NET* hospitalization data covers around 10% of the US population. Fourth, our model does not explicitly incorporate the individual level variations in health insurance coverage, which may impact hospitalization and ICU admission related to COVID-19. However, this aspect is implicitly encompassed through the probabilistic differences in hospitalization outcome across state of residence, age, and racial groups. Fifth, we combined the American Indian, Asian, Native Hawaiian/ Pacific Islander, multiracial and other races in one race category because of relatively lower sample size (across states) of these race categories in the BRFSS data. A disaggregated analysis of these race categories would have produced a more nuanced understanding of this critical public health issue.

We deliver a model that encompasses the differences in population level chronic condition prevalence across state, race, and age group to estimate COVID-19 related hospitalization and ICU admission outcomes. We, however, do not evaluate the interplays between socioeconomic factors, race and ethnicity concerning chronic condition prevalence and COVID-19 related hospitalization. The estimated differences in change in hospitalization and ICU admission rates across racial groups and geographic territories can be further investigated in future research. At the individual level, COVID-19 related hospitalization is also associated with financial burden and risk of economic hardship [33, 34]. Future research can explore the extent of economic burden that could have been avoided if the prevalence of chronic conditions were lower.

Due to data unavailability, we could not assess how better management of chronic conditions could impact hospitalization and subsequent ICU admissions of COVID-19 patients. For example, if we knew the share of individuals who have their hypertension under control in the status quo, then under appropriate assumptions, we could simulate alternate scenarios of enhanced hypertension control rate. Using fitting data, this can be studied in future research to showcase the importance of effective chronic condition management for preventing health system overwhelming during a pandemic.

## Conclusions

Our model provides evidence on the importance of chronic disease management at the population level in order to lessen the overwhelming burden of the health system during a pandemic. Apart from studying COVID-19 related mortality, a more objective outcome concerning population level underlying chronic conditions, we focus on the hospitalization and ICU admission, which are important indicators of health service delivery. We explore to what extent the population level chronic condition prevalence takes a toll on the health system capacity during the pandemic. The goal of this research is not to predict the number of hospitalization or ICU admission ex-ante, but to demonstrate how the numbers would have been lowered had there been a lower prevalence of the chronic condition (than status quo) in the population. The escalating number of COVID-19 related hospitalizations strains the health system and could have been significantly reduced by prevention and control of the major chronic conditions at the population level.

## Data Availability

The datasets generated and/or analysed during the current study are available in the National Vital Statistics System – Bridged-Race Population Estimates - Data Files and Documentation (https://www.cdc.gov/nchs/nvss/bridged_race/data_documentation.htm#vintage2019), COVIDView (https://www.cdc.gov/coronavirus/2019-ncov/covid-data/covidview/01042021/specimens-tested.html), CDC COVID Data Tracker (https://covid.cdc.gov/covid-data-tracker/), and Behavioral Risk Factor Surveillance System (https://www.cdc.gov/brfss/annual_data/annual_2019.html) repository.

## Abbreviations

CVD: Cardiovascular Disease
ICU: Intensive Care Unit
CDC: Centers for Disease Control and Prevention
NCDs: Non-communicable diseases
WHO: World Health Organization
